# Inhibition of Adipogenesis by Oligonol through Akt-mTOR Inhibition in 3T3-L1 Adipocytes

**DOI:** 10.1155/2014/895272

**Published:** 2014-09-11

**Authors:** Jae-Yeo Park, Younghwa Kim, Jee Ae Im, Seungkwon You, Hyangkyu Lee

**Affiliations:** ^1^Department of Clinical Nursing Science, Yonsei University College of Nursing, Seoul 120-752, Republic of Korea; ^2^Nursing Policy and Research Institute, Biobehavioral Research Center, Yonsei University, Seoul 120-752, Republic of Korea; ^3^Division of Biotechnology, College of Life Sciences and Biotechnology, Korea University, Seoul 136-701, Republic of Korea; ^4^Department of Emergency Medical Technology, Kyungil Univeristy, Gyeongsan, Kyungbook 712-701, Republic of Korea; ^5^Sport and Medicine Research Center, INTOTO Inc., 401 Dawoo BD, 90-6 Daeshin-Dong, Seodaemun-Gu, Seoul 120-160, Republic of Korea

## Abstract

Polyphenols have recently become an important focus of study in obesity research. Oligonol is an oligomerized polyphenol, typically comprised of catechin-type polyphenols from a variety of fruits, which has been found to exhibit better bioavailability and bioreactivity than natural polyphenol compounds. Here, we demonstrated that Oligonol inhibits 3T3-L1 adipocyte differentiation by reducing adipogenic gene expression. During adipogenesis, Oligonol downregulated the mRNA levels of peroxisome proliferator-activated receptor *γ* (PPAR*γ*), CCAAT/enhancer binding proteins *α* (C/EBP*α*), and *δ* (C/EBP*δ*) in a dose-dependent manner and the expression of genes involved in lipid biosynthesis. The antiadipogenic effect of Oligonol appears to originate from its ability to inhibit the Akt and mammalian target of rapamycin (mTOR) signaling pathway by diminishing the phosphorylation of ribosomal protein S6 kinase (p70S6K), a downstream target of mTOR and forkhead box protein O1 (Foxo1). These results suggest that Oligonol may be a potent regulator of obesity by repressing major adipogenic genes through inhibition of the Akt signaling pathway, which induces the inhibition of lipid accumulation, ultimately inhibiting adipogenesis.

## 1. Introduction

Obesity is rapidly becoming a global health problem and is a leading cause of metabolic syndrome, cardiovascular diseases, and type II diabetes. Obesity may be attributed to a number of factors, including changes in genetic predispositions, eating habits, and lack of exercise. One of the mechanisms proposed to explain the cause of obesity is an increase in adipose tissue resulting from an increase in the number of fat cells (adipocytes) through adipogenesis, leading to a subsequent increase in lipid accumulation in adipose tissue. Therefore, finding an effective strategy to suppress adipogenesis is of significant interest in the field of antiobesity research.

Adipogenesis involves the development of fat cells when undifferentiated preadipocytes are converted to fully differentiated, mature adipocytes. The mechanism of adipogenesis is tightly regulated by several key transcription factors. However, it is difficult to dissect the phases of adipogenesis in vivo due to heterogenic differentiation of adipose tissue, which occurs throughout the lifetime of an organism. The establishment of the 3T3-L1 cell line has provided us with an opportunity to study adipocyte differentiation. Although its morphology is indistinguishable from that of fibroblasts, this cell line is committed to the adipocyte lineage. Treatment with a mixture of insulin, dexamethasone, and 3-isobutyl-1-methylxanthine induces the adipogenic differentiation process in 3T3-L1 cells [1].

Peroxisome proliferator-activated receptor *γ* (PPAR*γ*) and CCAAT/enhancer binding proteins (C/EBP*α*, C/EBP*β*, and C/EBP*δ*) are key transcription factors in adipogenesis. C/EBP*β* and C/EBP*δ* are induced during the early phases of adipogenesis. Activation of these transcriptional components is then followed by activation of PPAR*γ* and C/EBP*α*, the central transcriptional regulators in adipogenesis. Once PPAR*γ* and C/EBP*α* are activated, they cooperatively enhance each other, which then induces further expression of adipogenic genes required for maintaining adipocyte characteristics and terminal differentiation [1, 2]. In particular, PPAR*γ* is a central regulator of adipogenesis and represents a good target for antiobesity therapy.

Flavonoids and polyphenols are natural chemical compounds derived from fruits and vegetables and are known to function as anti-inflammatory and chemopreventive agents on various human diseases. Both in vivo and in vitro studies have demonstrated that flavonoids and polyphenols can be used to treat neurological, metabolic, cardiovascular, and psychiatric diseases [3]. However, due to their high molecular weights, polyphenol polymers exhibit inefficient bioreactivity and poor bioabsorption. In order to overcome these limitations, a novel technology was used to optimize the oligomerization of polyphenol polymers. Oligonol is an oligomerized polyphenol, typically comprised of catechin-type polyphenols from a variety of fruits (e.g., grapes, apples, and persimmons). A typical polyphenol contains less than 10% oligomers, whereas Oligonol consists of more than 50% oligomers (i.e., monomers to pentamers) [3]. It is interesting to note that synthetic Oligonol has thus far exhibited better bioavailability than natural polyphenol compounds [4] and appears to be safe for human use at doses lower than 200 mg/day [5].

Several studies have reported the antiobesity effects of Oligonol. Oral administration of Oligonol decreases white adipose tissue mass and attenuates dysregulated expression of adipokines in adipose tissues of mice consuming high fat diets [6]. The same research group also reported that Oligonol enhances lypolysis in primary adipocytes through activation of the extracellular signaling-regulated kinases 1/2 (ERK1/2) pathway accompanied by downregulation of perilipin [7]. However, the potential antiobesity effects of Oligonol on adipogenesis are not known; therefore, the aim of this present study was to investigate whether Oligonol inhibits the adipogenic process using 3T3-L1 cells, a well-established model system, to elucidate the underlying molecular mechanisms of Oligonol-induced modification of in vitro adipocyte differentiation.

## 2. Materials and Methods

### 2.1. Materials

The 3T3-L1 cells were graciously provided by Dr. Jae-Woo Kim (Yonsei University, College of medicine). Oligonol was supplied by KCF Korea Co. (Seoul, Korea). The triglyceride quantification kit was purchased from BioVision (Mountain View, CA, USA). Antibodies for experiments are the following: CEBP*α*, pACC(Ser79), ACC, FAS, pAKT(Ser473), AKT, pFOXO1(Thr24), FOXO, pAMPK(Thr172), AMPK, pmTOR(Ser2448), mTOR, pS6K(Thr389), S6K, pS6 (Cell Signaling, Danvers, MA, USA), PPAR*γ*, GLUT4, Adiponectin (Santa Cruz Biotechnology, Santa Cruz, CA, USA), aP2 (Cayman, Ann Arbor, MI, USA), and *β*-actin (Sigma-Aldrich, Oakville, Canada).

### 2.2. Cell Culture

3T3-L1 preadipocytes were maintained in Dulbecco's modified eagle medium (DMEM) containing 10% calf serum at 37°C and 5% CO_2_. After reaching confluence, differentiation to adipocytes was induced by treatment with insulin, dexamethasone (DEX), and isobutylmethylxanthine (IBMX) in DMEM containing 10% FBS for 2 days (DM1). The medium was replaced with DMEM containing insulin for additional 2 days (DM2) and then replaced with fresh DMEM containing 10% FBS every other day. Oligonol was dissolved in ethanol, directly diluted in DMEM, and treated during the course of differentiation. The final concentration of the ethanol did not exceed 0.1% for either the control or the treated cells for all experiments.

### 2.3. MTT Assay

For initial screening of cytotoxicity, 3T3-L1 preadipocytes were grown in a 96-well plate with various concentrations of Oligonol for 24 hrs. After determination of cytotoxic dose of Oligonol, cells were treated with 10, 25, or 50 *μ*g/mL of Oligonol for 4 days after induction of differentiation (from day 4 to day 8), or for 8 days during entire experiments. Cell viability was determined colorimetrically using 3-(4,5-dimethylthiazol-2-yl)-2,5-diphenyltetrazolium bromide (MTT). Insoluble formazan crystals were dissolved in isopropanol, and absorbance was measured at 490 nm using a microplate reader (Molecular Devices, Mountain View, CA, USA).

### 2.4. Oil Red O Staining and Quantification

Fully differentiated 3T3-L1 cells were washed twice with PBS and fixed in 4% paraformaldehyde for 1 h. Cells were stained with 3 g/L of Oil Red O (Sigma Chemical, St. Louis, MO) in 60% isopropanol at room temperature for 10 min and washed extensively with distilled water. Pictures were taken using a microscope (Axiovert 40CFL; Olympus, Germany). In addition, stained Oil Red O dye was extracted with isopropanol and collected, and the absorbance (O.D. 500 nm) was measured by microplate reader (Versamax; Molecular Devices Corporation, CA, USA).

### 2.5. Quantitative Real-Time PCR

Total RNA from 3T3-L1 cells was isolated using an RNeasy mini kit (Qiagen, Hilden, Germany). Aliquots of 2 *μ*g of total RNA were used to synthesize cDNA using the QuantiTech Reverse transcription kit (Qiagen, Hilden, Germany) according to the manufacturer's instructions. After reverse transcription, samples were analyzed by SYBR premix Ex Taq using Takara Thermal Cycler dice real-time system (Otsu, Shiga, Japan).

Each cDNA was amplified (95°C for 5 s, 58–64°C for 10 s, and 72°C for 20 s for 40 cycles) using specific primers designed from sequences derived from the NCBI nucleotide sequence database (Table 1). All reactions were performed in triplicate, and the data were normalized to GAPDH as an internal control.

### 2.6. Western Blot Analysis

Cells were extracted in the extraction buffer (50 mM Tris HCl, pH 8.0, 5 mM EDTA, 150 mM NaCl, 0.5% sodium deoxycholate, 1% Nonidet P-40, 0.1% SDS, 1 mM PMSF, 1 mM NaF, 1 mM NaVO_4_, and protease inhibitor cocktails (Roche, Germany)). Equal amounts of protein extracts were separated on 10% polyacrylamide gels and electrophoretically transferred onto polyvinylidene fluoride membrane (Gelman Laboratory, MI, USA). After blocking, the membranes were incubated with each primary antibody and then with HRP-conjugated IgG (Santa Cruz Biotechnology, Santa Cruz, CA, USA). The blots were developed using an ECL detection kit.

### 2.7. Triglyceride Assay

Triglyceride content was determined using an EnzyChrom Triglyceride Assay kit (BioAssays, Hayward, CA, USA) according to the manufacturer's instructions. Briefly, Oligonol was used to treat 3T3-L1 adipocytes 8 days after their differentiation. For cellular triglyceride analysis, cells were washed with PBS and solubilized in 5% Triton X-100. Colorimetric intensity was determined at 570 nm, and quantification was performed using a 96-well plate reader (Molecular Devices, Mountain View, USA).

### 2.8. Glycerol Assay

3T3-L1 adipocytes were fully differentiated for 8 days and then treated with different concentration of Oligonol for 24 and 48 hrs. After treatment of differentiated 3T3-L1 cells with Oligonol, free glycerol contents in the cell supernatants were quantified using a glycerol quantification kit according to the manufacturer's instructions (Biovision Inc., Milpitas, USA). Glycerol was quantified at 570 nm on a 96-well plate reader (Molecular devices, Mountain View, USA).

### 2.9. Statistics

The data are shown as mean ± SEM. Differences between means of each group were analyzed using Student's *t*-test or one-way ANOVA test with Dunnett's multiple comparison test. *P* values of <0.05 were considered significant. The statistical software package Prism 5.0 (GraphPad Software, La Jolla, CA, USA) was used for the analysis.

## 3. Results

### 3.1. Effect of Oligonol on 3T3-L1 Differentiation

**Figure 1:**
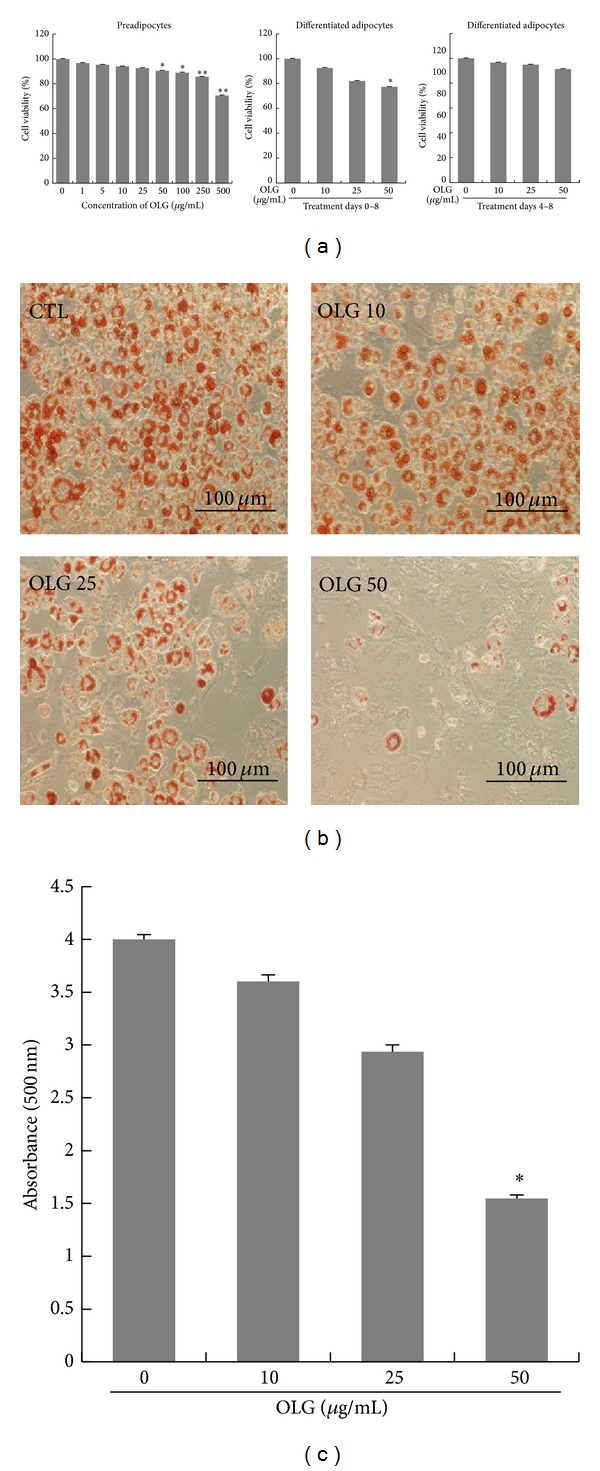
Effect of Oligonol on 3T3-L1 adipocytes. (a) Cell viability of 3T3-L1 preadipocytes and differentiated adipocytes treated with Oligonol (OLG). 3T3-L1 preadipocytes were incubated with various concentrations of Oligonol (0–500 *μ*g/mL) for 24 hrs and cell viability was measured by MTT assay. 3T3-L1 preadipocytes were differentiated into adipocytes with the MDI (mixture of IBMX, dexamethasone, and insulin)—containing media for 8 days. Cells were treated with 10, 25, or 50 *μ*g/mL of Oligonol during entire differentiation period (from day 0 to day 8) or after induction of differentiation (from day 4 to day 8). (b) Lipid accumulation was determined by Oil Red O staining. (c) Cellular lipid accumulation was quantified by measuring the absorbance at 500 nm. Values are mean ± SEM of three independent experiments carried out in triplicates. ∗*P* < 0.05, ∗∗*P* < 0.01 compared with untreated control.

To test any possible toxic effects of Oligonol on 3T3-L1 cells, we evaluated cell viability and cytotoxicity using the MTT assay. 3T3-L1 cells were incubated with various concentrations of Oligonol for 24 hrs, and the percentage of cell viability was determined compared to control cells (set as 100%). We observed 90% or higher cell viability when 3T3-L1 cells were treated with up to 250 *μ*g/mL of Oligonol for 24 hrs. Since mild toxicity during adipocyte differentiation was observed with Oligonol concentrations exceeding 250 *μ*g/mL, subsequent experiments were conducted using Oligonol at concentrations less than or equal to 50 *μ*g/mL. When 3T3-L1 cells were treated with Oligonol for 8 days during adipocyte differentiation, 50 *μ*g/mL of Oligonol showed mild inhibitory effect on cell viability on day 8 by MTT assay; however, there was no sign of cytotoxicity or cell death, and cells were morphologically similar to preadipocytes. When cells were treated with the same doses of Oligonol from day 4 to day 8 after induction of differentiation with differentiation cocktail (insulin, DEX, and IBMX) treatment with Oligonol did not show any significant inhibitory effect on cell viability in adipocytes (Figure 1(a)).

Based on previous reports indicating the beneficial effects of polyphenol compounds for treating obesity, we investigated whether Oligonol inhibits adipocyte differentiation. 3T3-L1 preadipocytes were maintained in a differentiated medium for 8 days in the absence or presence of 10, 25, or 50 *μ*g/mL of Oligonol. Fully differentiated 3T3-L1 adipocytes were stained, and total lipid accumulation was quantified by Oil Red O. Fully differentiated adipocytes are characterized by lipid droplets filling the cytoplasmic space of the cells. In contrast to the control cells, cells incubated with Oligonol showed reduced numbers of lipid droplet—containing cells in a dose—dependent manner, and the few vesicles detected under a microscope were smaller in size (Figure 1(b)). Oligonol also significantly reduced total cytosolic lipid content compared to control cells (Figure 1(c)), which was in agreement with observations from phase contrast microscopy (Figure 1(b)). It was notable that substantial decrease of lipid accumulation was not due to cytotoxic effect, because both control cells and drug-treated cells were grown in comparable cell densities. Oligonol treated cells maintained fibroblast-like preadipocyte appearance without disrupting the integrity of a cell monolayer as monitored under microscope during adipocyte differentiation. These results demonstrated that Oligonol prevented adipocyte differentiation in 3T3-L1 without affecting cell growth or viability.

### 3.2. Effect of Oligonol on the Expression of Adipocyte-Specific Transcription Factors during 3T3-L1 Differentiation

Adipocyte differentiation is accompanied by the increased expression of various adipocyte-associated transcription factors. To investigate the mechanisms underlying Oligonol-induced inhibition of 3T3-L1 differentiation in detail, we first examined which transcription factors could be affected by Oligonol. Transcription factors, such as C/EBP*α*, C/EBP*β*, C/EBP*δ*, and PPAR*γ*, are known to be key markers in the adipogenic process, especially during the early and middle stages of adipogenesis [8]. These transcription factors are highly induced during adipogenesis; therefore, we treated 3T3-L1 cells in the process of differentiation with various concentrations of Oligonol, and determined the gene expression of each transcription factor by quantitative RT-PCR.

**Figure 2:**
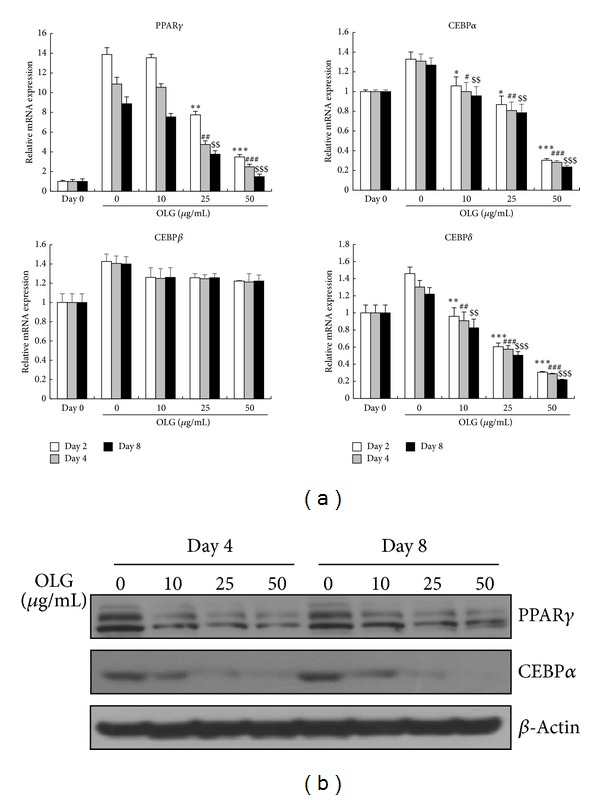
Effect of Oligonol on the expression of adipocyte-specific transcription factors during 3T3-L1 adipocyte differentiation. 3T3-L1 preadipocytes were differentiated into adipocytes in the absence or presence of Oligonol (10, 25, or 50 *μ*g/mL) for 8 days. (a) Total RNA was isolated from 3T3-L1 adipocytes at days 2, 4, and 8 during differentiation. mRNA expressions of PPAR*γ*, C/EBP*α*, C/EBP*β*, and C/EBP*δ* were analyzed by quantitative RT-PCR. All gene expressions were normalized using GAPDH as a reference gene. (b) Protein expression levels of PPAR*γ* and C/EBP*α* were analyzed by Western blot analysis. Cell lysates were collected from 3T3-L1 cells at days 4 and 8 after induction of adipocyte differentiation. Values are mean ± SEM of three independent experiments carried out in triplicates. ∗*P* < 0.05, ∗∗*P* < 0.01, ∗∗∗*P* < 0.005 compared with untreated adipocytes at day 2. ^#^ *P* < 0.05, ^##^ *P* < 0.01, ^###^ *P* < 0.005 compared with untreated adipocytes at day 4. ^$^ *P* < 0.05, ^$$^ *P* < 0.01, ^$$$^ *P* < 0.005 compared with untreated adipocytes at day 8 at each gene expression.

mRNA levels of the transcription factors PPAR*γ*, C/EBPs were significantly increased at day 2, and gradually declined at days 4 and 8 during adipocyte differentiation. Treatment of Oligonol reduced mRNA levels of PPAR*γ*, C/EBP*α*, and C/EBP*δ* relative to untreated cells in a dose-dependent manner, whereas mRNA levels of C/EBP*β* were not affected by Oligonol (Figure 2(a)). Protein levels of PPAR*γ* and C/EBP*α* were also confirmed by Western blot analysis. As expected, PPAR*γ* and C/EBP*α* were highly induced during adipocyte differentiation, and Oligonol significantly inhibited protein levels of both transcription factors in a dose-dependent manner. Inhibitory effect of Oligonol was maintained until day 8, the termination stage of 3T3-L1 differentiation (Figure 2(b)).

### 3.3. Effect of Oligonol on the Expression of Adipogenesis Markers

**Figure 3:**
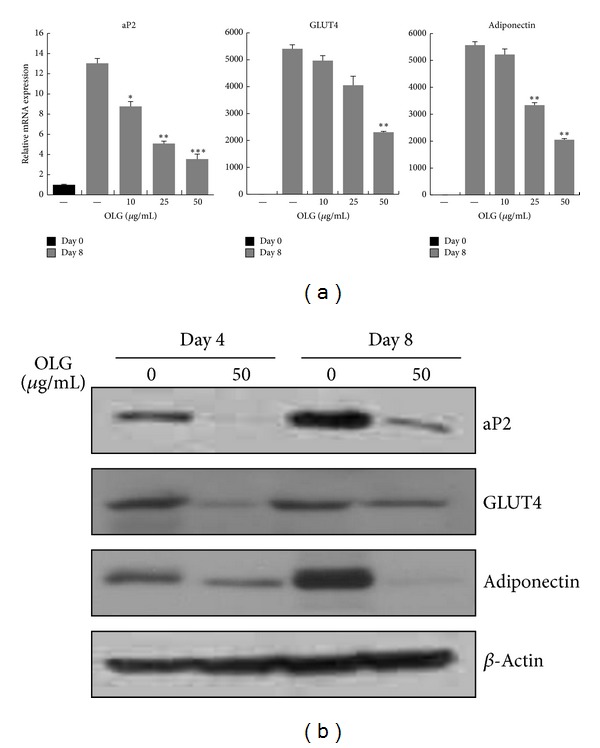
Effect of Oligonol on the expression of adipogenesis-related genes during 3T3-L1 adipocyte differentiation. 3T3-L1 preadipocytes were differentiated into adipocytes in the absence or presence of Oligonol (10, 25, or 50 *μ*g/mL) for 8 days. (a) Oligonol inhibited the mRNA expressions of aP2, GLUT4, and Adiponectin during differentiation. The gene expressions were analyzed by quantitative RT-PCR. All gene expressions were normalized using GAPDH as a reference gene. (b) Protein expression levels of aP2, GLUT4, and adiponectin were analyzed by Western blot analysis in 3T3-L1 adipocytes treated with Oligonol. Cell lysates were collected from 3T3-L1 cells at days 4 and 8 after induction of adipocyte differentiation. Values are mean ± SEM of three independent experiments carried out in triplicates. ∗*P* < 0.05, ∗∗*P* < 0.01, ∗∗∗*P* < 0.005 compared with untreated adipocytes at each gene expression.

Since Oligonol exhibited antiadipogenic effects in the early stage, we hypothesized that the target gene expression of those transcription factors might be downregulated. Indeed, the mRNA levels of markers associated with the final stage of adipogenesis, including adipocyte protein 2 (aP2), glucose transporter 4 (GLUT4), and adiponectin, were significantly reduced by Oligonol (Figure 3(a)). Western blot analysis confirmed the expression levels of adipogenesis-associated genes at the protein level. Consistent with the results in Figure 2, protein expressions of aP2, GLUT4, and adiponectin were significantly reduced in 3T3-L1 cells by continuous treatment of Oligonol (Figure 3(b)).

### 3.4. Effect of Oligonol on the Expression of Lipogenic Genes and Lipid Accumulation

**Figure 4:**
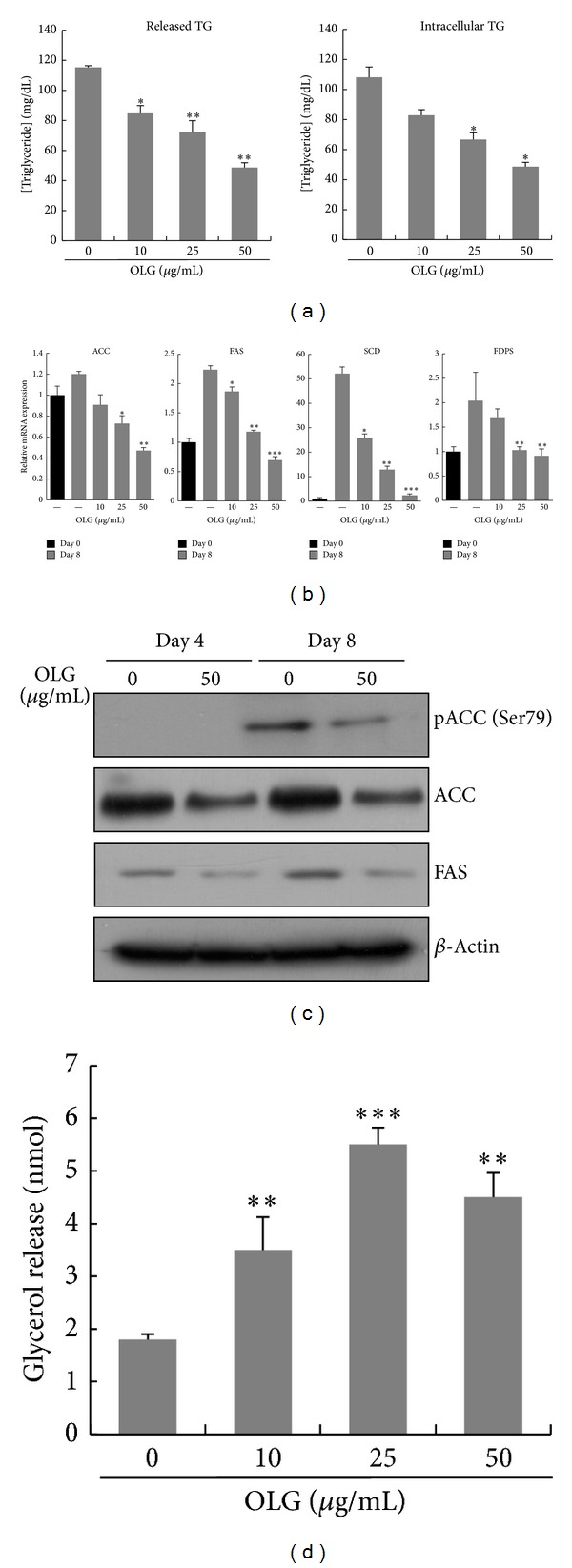
Effect of Oligonol on lipid biosynthesis and lipolysis in 3T3-L1 adipocytes. 3T3-L1 preadipocytes were differentiated into adipocytes with the absence or presence of Oligonol (10, 25, or 50 *μ*g/mL) for 8 days. (a) Oligonol reduced TG content during differentiation of 3T3-L1 adipocyte. 3T3-L1 preadipocytes were differentiated in the absence or presence of Oligonol for 8 days, and the lipid accumulation was measured by triglyceride assay. (b) Oligonol inhibited the mRNA expressions of lipogenic gene, ACC, FAS, and SCD during differentiation. The gene expressions were analyzed by quantitative RT-PCR. All gene expressions were normalized using GAPDH as reference gene. (c) Protein expression levels of phospho-ACC, ACC, and FAS were analyzed by Western blot analysis in 3T3-L1 adipocytes treated with Oligonol. Cell lysates were collected from 3T3-L1 cells at days 4 and 8 after induction of adipocyte differentiation. (d) Glycerol release into the medium was quantified in fully differentiated 3T3-L1 adipocytes treated with Oligonol (10, 25, or 50 *μ*g/mL). Values are mean ± SEM of three independent experiments carried out in triplicates. ∗*P* < 0.05, ∗∗*P* < 0.01, ∗∗∗*P* < 0.005 compared with untreated adipocytes.

During adipocyte differentiation, fibroblast-like preadipocytes convert to a spherical shape and accumulate lipid droplets [2]. Because Oligonol significantly inhibited the accumulation of lipid droplet in 3T3-L1 adipocytes, we evaluated whether Oligonol could inhibit the synthesis of fats by measuring triglyceride contents. 3T3-L1 preadipocytes were induced into differentiation in the absence or presence of Oligonol, and triglyceride content was quantified on day 8 of the differentiation periods. As seen in Figure 4(a), 3T3-L1 adipocytes cultured in a medium containing Oligonol displayed reduced intracellular triglyceride content in a dose-dependent manner. The release of triglycerides into the conditioning media was also reduced.

Reduction of triglyceride content was observed in the cells treated with Oligonol, suggesting a role for Oligonol in lipogenesis. Acetyl-CoA carboxylase (ACC) and fatty acid synthase (FAS) are the primary enzymes involved in lipogenesis, and the genes encoding them are the target genes of PPAR*γ* and C/EBP*α*. At day 8, the mRNA levels of ACC and FAS were found to increase in fully differentiated 3T3-L1 adipocytes, whereas their mRNA levels were decreased with increasing dose of Oligonol in 3T3-L1 adipocytes. In addition, Oligonol also reduced the mRNA expression of other genes that are involved in lipid biosynthesis, such as stearoyl CoA-desaturase (SCD1) and farnesyl diphosphate synthase (FDPS) (Figure 4(b)). Western blot analysis also reconfirmed that Oligonol reduced the protein levels of phospho-ACC (Ser79) and FAS in 3T3-L1 adipocytes demonstrating that Oligonol has an antilipogenic effect on 3T3-L1 cells (Figure 4(c)).

### 3.5. Effect of Oligonol on Lipolysis in 3T3-L1 Adipocytes

We also extended our investigation of Oligonol on antiobesity effects by quantifying lipolysis of 3T3-L1 adipocytes. To examine whether Oligonol stimulated the lipolysis of 3T3-L1 adipocytes, cells were fully differentiated for 8 days and treated with Oligonol for the next 48 hrs. Free glycerol contents in a conditioning medium were measured as an indicator of lipolysis. As shown in Figure 4(d), Oligonol increased the significant amount of glycerol release. Glycerol release was higher in Oligonol treated 3T3-L1 cells at as low as 10 *μ*g/mL, and maximal glycerol release was observed at 25 *μ*g/mL of Oligonol concentration.

### 3.6. The Effect of Oligonol on the Regulation of the Akt Signaling Pathway during 3T3-L1 Differentiation

**Figure 5:**
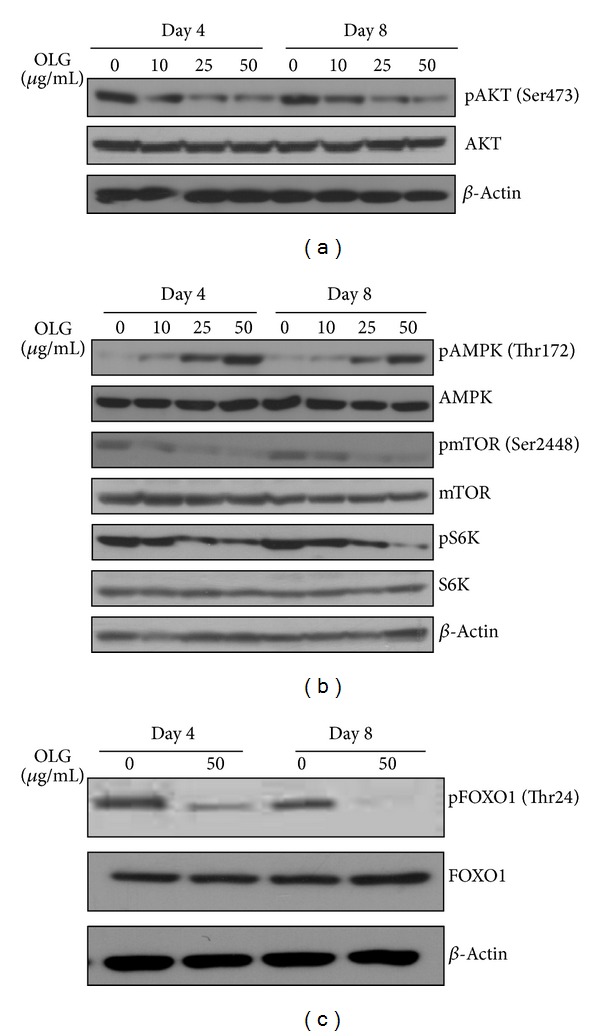
Effect of Oligonol on phosphorylation of AKT, mTOR, and Foxo1 in 3T3-L1 adipocytes. 3T3-L1 preadipocytes were differentiated into adipocytes with the absence or presence of Oligonol (10, 25, or 50 *μ*g/mL) for 8 days. (a) Oligonol decreased the serine phosphorylation of Akt in 3T3-L1 adipocytes. (b) Oligonol phosphorylated AMPK in a dose-dependent manner, and inversely attenuated the phosphorylation of mTOR-S6K in 3T3-L1 adipocytes as compared to untreated adipocytes. (c) Oligonol decreased the serine phosphorylation of FOXO1 in 3T3-L1 adipocyte. Cell lysates were collected from 3T3-L1 cells at days 4 and 8 after induction of adipocyte differentiation. Western blot analysis was performed using antibodies specific for the proteins indicated. The data shown are representative of three independent experiments.

We then investigated the state of the serine/threonine kinase Akt activation during 3T3-L1 adipogenesis and determined the effect of Oligonol on the molecular pathway triggered by Akt. Cell lysates were prepared from days 4 and 8 after initiation of differentiation and immunoblotted with antibodies against phosphor-Akt (Ser473) and total Akt. During normal differentiation with 3T3-L1 cells, we observed an increased level of Akt phosphorylation, which reflects Akt's enzymatic activity. In the presence of Oligonol, the expression of phospho-Akt was reduced without affecting total Akt protein expression in a dose-dependent manner (Figure 5(a)). In addition, the pattern of phospho-Akt expression correlated with PPAR*γ* and C/EBP*α* expression (Figure 2(b)).

Activation of Akt regulates a diverse array of biological processes [9], many of which could contribute to the role of Akt in driving adipogenesis. Mammalian target of rapamycin (mTOR) is a critical downstream signaling protein that promotes both adipogenesis and lipogenesis [10] by controlling PPAR*γ* and sterol regulatory element-binding protein-1 (SREBP-1). On the basis of the result that Oligonol inhibited Akt activation, we hypothesized that mTOR signaling pathway could be suppressed in 3T3-L1 adipocytes under Oligonol treatment. Similar to the result of Akt inactivation, the phosphorylation of mTOR was reduced in 3T3-L1 cells in the presence of Oligonol. This change was paralleled by the reduction of downstream signaling proteins, phosphoribosomal protein S6 kinase (S6K), ribosomal protein S6 (S6P), and transcription factor SREBP-1 expression, whereas AMP-activated protein kinase (AMPK) was phosphorylated by Oligonol treatment (Figure 5(b)).

We also investigated the phosphorylation of the transcription factor forkhead box protein O1 (Foxo1) in 3T3-L1 adipocytes treated with Oligonol. To investigate the regulation of Foxo1 in adipocytes, we performed Western blotting with a phospho-specific antibody against Thr24, the site for sufficient nuclear exclusion of Foxo1 [11, 12]. Foxo1 phosphorylation was detectable and remained until day 8 through the course of adipocyte differentiation. However, Oligonol significantly decreased the Foxo1 phosphorylation in agreement with Akt inactivation (Figure 5(c)). These results demonstrate that Oligonol suppresses the Akt activation, which leads to inhibition of its substrate Foxo1 phosphorylation.

## 4. Discussion

In this study, we investigated the effect of Oligonol on adipocyte differentiation using mouse 3T3-L1 cells as a model system. Oligonol inhibited the differentiation of 3T3-L1 preadipocytes into adipocytes, and our data suggest that this effect is through inhibition of the ATK-mTOR pathway, triglyceride accumulation, and lipogenesis.

Adipocyte differentiation is mediated by sequential activation of a complex transcriptional cascade. In response to an adipogenic induction, 3T3-L1 preadipocytes undergo mitotic clonal expansion by dramatic induction of C/EBP*β* and C/EBP*δ* at the first stage of adipogenesis. Then, C/EBP*β* and C/EBP*δ* promote the expression PPAR*γ* and C/EBP*α*, the central transcription factors in adipocyte differentiation. PPAR*γ* and C/EBP*α* cross-regulate each other through a positive feedback loop and act synergistically to promote terminal differentiation by transactivating target genes, such as aP2, GLUT4, and adiponectin, which are commonly used as adipocyte markers [2].

Oligonol is a lychee fruit-derived low-molecular form of polyphenol containing catechin-type monomers and lower oligomers of proanthocyanidin [13]. It is widely accepted that many polyphenols produce antiobesity effects through different targets. For instance, epigallocatechin-3-gallate (ECCG, a flavonoid from tea extract) has been shown to reduce mRNA expression of PPAR*γ* and C/EBP*α* without affecting C/EBP*β* or C/EBP*δ* [14], whereas* Citrus aurantium* flavonoids reduced the expression of C/EBP*β*, C/EBP*α*, and *α*PPAR*γ* [15]. Genistein (a soy-derived isoflavone) and resveratrol (a stilbenoid from grapes) block adipogenesis by targeting adipogenesis-associated transcription factors [16], while lycopene (a carotenoid in tomato) does not modulate adipogenesis in 3T3-L1 but prevents obesity-induced metabolic syndrome through regulation of adipokines and inflammatory responses [6, 17]. Furthermore, Oligonol has a better lipolytic effect than ECCG, and combined treatment of both drugs increases lipolytic effect [18]. These results support the notion that mixture of several polyphenol compounds helps to reduce obesity more effectively because each compound targets different molecules and increases a synergistic antiobesity effect.

To determine the stage of differentiation during which Oligonol inhibits adipogenesis, we investigated the expression levels of the critical transcription factors PPAR*γ*, C/EBP*α*, and C/EBP*β* isoforms. Our results indicated that mRNA expression of PPAR*γ* and C/EBP*α* was downregulated by Oligonol, whereas mRNA expression of C/EBP*β* was not affected (Figure 1). Microscopic inspection and Oil Red O staining also revealed that Oligonol treatment remarkably reduced lipid accumulation in 3T3-L1 cells and maintained the cells in a preadipocyte phenotype. Additionally, Oligonol significantly reduced GLUT4 and adiponectin expression compared to that in fully differentiated adipocytes (Figure 3(b)). These results indicated that Oligonol inhibited the differentiation of 3T3-L1 cells at the early phase of adipogenesis by repressing the expression of the master regulators PPAR*γ* and C/EBP*α* during adipocyte differentiation. Decreased lipid content and reduction of lipogenic gene expression were an obvious consequence of antiadipogenic effect of Oligonol in fully differentiated adipocytes.

It is noteworthy that Oligonol increases lipolysis. Previously, Oligonol has been shown to decrease the epididymal white adipose tissue mass of high fat diet-fed mice [6] and to enhance lypolysis in primary adipocytes through activation of the ERK1/2 pathway [7]. Consistent with those reports, treatment of Oligonol increased the glycerol release from fully differentiated 3T3-L1 adipocytes (Figure 4). Our study mainly focused on the effect of Oligonol on inhibition of adipogenesis; however, a concomitant lipolytic effect of Oligonol suggests that Oligonol can be developed as a dual antiobesity therapeutic agent.

Insulin is an essential ingredient for inducing adipogenesis. The Akt pathway is a downstream function of insulin signaling that is critically involved in adipocyte differentiation. RNAi or knockout for Akt gene in mouse model displays severe defects in adipocyte differentiation [19, 20], whereas overexpression of constitutively active Akt enhanced adipocyte differentiation and glucose uptake in 3T3-L1 preadipocytes [21]. Moreover, Akt activation inhibits downstream substrates such as GSK3*β* and Foxo1, which directly regulate PPAR*γ*, C/EBP*β*, and C/EBP*α* [22, 23]. The fact that Oligonol markedly inhibited Akt activation (Figure 5) strongly suggest that this may be the underlying molecular mechanism through which Oligonol inactivates PPAR*γ* and C/EBP*α* during early phase in 3T3-L1 adipocyte differentiation.

Adipocyte differentiation is also closely linked to the mammalian target of rapamycin (mTOR) pathway. It has been previously demonstrated that treatment with rapamycin (also known as sirolimus) during 3T3-L1 adipocyte differentiation resulted in the reduction of both mRNA and protein levels of PPAR*γ* and C/EBP*α*, thereby indicating that mTOR inactivation prevents adipocyte differentiation [24]. As PPAR*γ* and C/EBP*α* appear to be specific target transcription factors of Oligonol, we tested the effect of Oligonol on the mTOR pathway in 3T3-L1 cells. Oligonol attenuated phosphorylation of both mTOR and the downstream substrate p70S6K (Figure 5), suggesting that the action of Oligonol is similar to that of rapamycin in 3T3-L1. Prolonged treatment of rapamycin inhibits adipocyte differentiation in 3T3-L1 cells [25, 26] and in human primary adipocytes [19]. One the other hand, AMPK, an upstream molecule of mTOR, was phosphorylated by Oligonol treatment. Growing evidences reported that AMPK is a new molecular target of various phytochemicals such as curcumin, flavonoids, and polyphenols in the prevention and treatment of cancers [27]. Anthocyanin, a flavonoid, was demonstrated to activate AMPK, leading to reduction in mTOR phosphorylation and inhibiting cancer cell growth [28]; Fisetin induces apoptosis by activating AMPK and suppressing mTOR in non-small lung cancer [29] and prostate cancer cells [30]. In another study, curcumin stimulates AMPK activation resulting in downregulation of PPAR*γ* in 3T3-L1 cells and in COX-2 in MCF-7 breast cancer cells [31]. Moreover, synthetic AMPK activator also supported the evidence that AMPK regulates signal of PPAR*γ* in 3T3-L1 adipocytes. Further studies will be necessary, but it is very likely that Oligonol blocks the activation of the Akt-mTOR signaling cascade, which then subsequently suppresses the adipogenic transcription factors PPAR*γ* and C/EBP*α*.

Although mTOR appears to be a good target for antiobesity therapy, conflicting clinical cases have reported problems associated with mTOR inhibition. For example, rapamycin is a widely used immunosuppressant used to prevent graft rejection in organ transplantation. One of the major side effects associated with rapamycin is dyslipidemia characterized by elevating serum cholesterol and triglycerides levels [32, 33]. Transplantation recipients receiving extended treatment with rapamycin also exhibit diabetes-like symptoms, including reduced glucose tolerance and insulin resistance [34]. Such adverse effects are potentially caused by the different functions of two different complexes of mTOR, mTORC1, and mTOR2, in the liver [35]. However, very little is known regarding the mechanism through which deregulation of lipid metabolism occurs. mTOR-mediated antiadipogenic effect of Oligonol in 3T3-L1 cells may suggest a tissue-specific function of the mTOR pathway in lipid homeostasis. On the other hand, lipolytic effect of Oligonol [7, 18] suggests that it performs a second function as an antiobesity reagent through inhibition of adipogenesis and activation of lipolysis. Our observation that Oligonol inhibits angiogenesis in adipocytes will provide a rationale for evaluating Oligonol as a potential antiobesity reagent in vivo.

## 5. Conclusion

The aims of this study were to investigate a potential antiadipogenic effect of Oligonol and to explore the underlying molecular mechanisms on adipocyte differentiation. We showed that Oligonol significantly suppressed adipogenic process by inhibiting the expression of C/EBP*α*, C/EBP*δ*, and PPAR*γ* and reducing lipid accumulation in 3T3-L1 adipocytes. Oligonol inhibited protein phosphorylation of Akt and the downstream signaling proteins mTOR and p70S6K during adipocytes differentiation. Therefore, we propose that Oligonol inhibits the Akt-mTOR pathway in 3T3-L1 cells, resulting in the prevention of adipogenesis at an early stage of adipocyte differentiation. These results suggest that Oligonol has a beneficial effect on prevention and treatment of obesity.

## Figures and Tables

**Table 1:** Sequence of primers used for RT-PCR.

Genes	Forward primer	Reveres primer	Tm (°C)
PPAR*γ*	CTTGTGAAGGATGCAAGGGT	ATACAAATGCTTTGCCAGGG	62
C/EBP*α*	TTGAAGCACAATCGATCCATCC	GCACACTGCCATTGCACAAG	64
C/EBP*β*	ACCGGGTTTCGGGACTTGA	CCCGCAGGAACATCTTTAAGTGA	58
C/EBP*δ*	TGATCTGCACGGCCTGTTGTA	GCACTTTGGGCAGGGATTTG	60
aP2	TGGGAACCTGGAAGCTTGTCTC	GAATTCCACGCCCAGTTTGA	60
GLUT4	GCTTTGTGGCCTTCTTTGAG	CAGGAGGACGGCAAATAGAA	60
Adiponectin	GAAGATGACGTTACTACAAC	TCAGTTGGTATCATGGAAGA	58
ACC	GAAGTCAGAGCCACGGCACA	GGCAATCTCAGTTCAAGCCAGTC	62
FAS	AGCACTGCCTTCGGTTCAGTC	AAGAGCTGTGGAGGCCACTTG	62
SCD	TCTTGTCCCTATAGCCCAATCCAG	AGCTCAGAGCGCGTGTTCAA	62
FDPS	ACACGCCAATGCCCTGAAG	AGCCAGCTGCATTTGTTGTCC	62
GAPDH	TGAACGGGAAGCTCACTGG	TCCACCACCCTGTTGCTGTA	58–64

## References

[1] Cao Z, Umek RM, McKnight SL (1991). Regulated expression of three C/EBP isoforms during adipose conversion of 3T3-L1 cells. *Genes and Development*.

[2] Gregoire FM (2001). Adipocyte differentiation: from fibroblast to endocrine cell. *Experimental Biology and Medicine*.

[3] Aruoma OI, Sun B, Fujii H, et al (2006). Low molecular proanthocyanidin dietary biofactor Oligonol: Its modulation of oxidative stress, bioefficacy, neuroprotection, food application and chemoprevention potentials. *BioFactors*.

[4] Fujii H, Yokozawa T, Kim YA, Tohda C, Nonaka G-I (2006). Protective effect of grape seed polyphenols against high glucose-induced oxidative stress. *Bioscience, Biotechnology and Biochemistry*.

[5] Fujii H, Sun B, Nishioka H, Hirose A, Aruoma OI (2007). Evaluation of the safety and toxicity of the oligomerized polyphenol Oligonol. *Food and Chemical Toxicology*.

[6] Sakurai T, Nishioka H, Fujii H (2008). Antioxidative effects of a new lychee fruit-derived polyphenol mixture, oligonol, converted into a low-molecular form in adipocytes. *Bioscience, Biotechnology and Biochemistry*.

[7] Ogasawara J, Kitadate K, Nishioka H (2009). Oligonol, a new lychee fruit-derived low-molecular form of polyphenol, enhances lipolysis in primary rat adipocytes through activation of the ERK1/2 pathway. *Phytotherapy Research*.

[8] Gregoire FM, Smas CM, Sul HS (1998). Understanding adipocyte differentiation. *Physiological Reviews*.

[9] Manning BD, Cantley LC (2007). AKT/PKB Signaling: navigating Downstream. *Cell*.

[10] Thirunavukkarasu M, Zhan L, Wakame K, Fujii H, Moriyama H, Bagchi M (2012). Safety of oligonol, a highly bioavailable lychee-derived polyphenolic antioxidant, on liver, kidney and heart function in rats. *Toxicology Mechanisms and Methods*.

[11] Zhao X, Gan L, Pan H (2004). Multiple elements regulate nuclear/cytoplasmic shuttling of FOXO1: characterization of phosphorylation- and 14-3-3-dependent and -independent mechanisms. *Biochemical Journal*.

[12] Guo H, Xia M, Zou T, Ling W, Zhong R, Zhang W (2012). Cyanidin 3-glucoside attenuates obesity-associated insulin resistance and hepatic steatosis in high-fat diet-fed and db/db mice via the transcription factor FoxO1. *Journal of Nutritional Biochemistry*.

[13] Fujii H, Nishioka H, Wakame K, Magnuson BA, Roberts A (2008). Acute, subchronic and genotoxicity studies conducted with Oligonol, an oligomerized polyphenol formulated from lychee and green tea extracts. *Food and Chemical Toxicology*.

[14] Söhle J, Knott A, Holtzmann U (2009). White Tea extract induces lipolytic activity and inhibits adipogenesis in human subcutaneous (pre)-adipocytes. *Nutrition and Metabolism*.

[15] Kim GS, Park HJ, Woo JH (2012). Citrus aurantium flavonoids inhibit adipogenesis through the Akt signaling pathway in 3T3-L1 cells. *BMC Complementary and Alternative Medicine*.

[16] Andersen C, Rayalam S, Della-Fera MA, Baile CA (2010). Phytochemicals and adipogenesis. *BioFactors*.

[17] Gouranton E, Aydemir G, Reynaud E (2011). Apo-10′-lycopenoic acid impacts adipose tissue biology via the retinoic acid receptors. *Biochimica et Biophysica Acta*.

[18] Ogasawara J, Kitadate K, Nishioka H (2011). Comparison of the effect of oligonol, a new lychee fruit-derived low molecular form of polyphenol, and epigallocatechin-3-gallate on lipolysis in rat primary adipocytes. *Phytotherapy Research*.

[19] Baudry A, Yang Z-Z, Hemmings BA (2006). PKB*α* is required for adipose differentiation of mouse embryonic fibroblast. *Journal of Cell Science*.

[20] Zhang HH, Huang J, Düvel K (2009). Insulin stimulates adipogenesis through the Akt-TSC2-mTORC1 pathway. *PLoS ONE*.

[21] Xu J, Liao K (2004). Protein kinase B/AKT 1 plays a pivotal role in insulin-like growth factor-1 receptor signaling induced 3T3-L1 adipocyte differentiation. *Journal of Biological Chemistry*.

[22] Grimes CA, Jope RS (2001). The multifaceted roles of glycogen synthase kinase 3*β* in cellular signaling. *Progress in Neurobiology*.

[23] Ross SE, Erickson RL, Hemati N, MacDougald OA (1999). Glycogen synthase kinase 3 is an insulin-regulated C/EBP*α* kinase. *Molecular and Cellular Biology*.

[24] Kim JE, Chen J (2004). Regulation of peroxisome proliferator-activated receptor-*γ* activity by mammalian target of rapamycin and amino acids in adipogenesis. *Diabetes*.

[25] Bell A, Grunder L, Sorisky A (2000). Rapamycin inhibits human adipocyte differentiation in primary culture. *Obesity Research*.

[26] Yeh WC, Bierer BE, McKnight SL (1995). Rapamycin inhibits clonal expansion and adipogenic differentiation of 3T3-L1 cells. *Proceedings of the National Academy of Sciences of the United States of America*.

[27] Kim I, He YY (2013). Targeting the AMP-activated protein kinase for cancer prevention and therapy. *Frontiers in Oncology*.

[28] Lee YK, Lee WS, Kim GS, Park OJ (2010). Anthocyanins are novel AMPK*α*1 stimulators that suppress tumor growth by inhibiting mTOR phosphorylation. *Oncology Reports*.

[29] Khan N, Afaq F, Khusro FH, Mustafa Adhami V, Suh Y, Mukhtar H (2012). Dual inhibition of phosphatidylinositol 3-kinase/Akt and mammalian target of rapamycin signaling in human nonsmall cell lung cancer cells by a dietary flavonoid fisetin. *International Journal of Cancer*.

[30] Suh Y, Afaq F, Khan N, Johnson JJ, Khusro FH, Mukhtar H (2010). Fisetin induces autophagic cell death through suppression of mTOR signaling pathway in prostate cancer cells. *Carcinogenesis*.

[31] Lee YK, Lee WS, Hwang JT, Kwon DY, Surh YJ, Park OJ (2009). Curcumin exerts antidifferentiation effect through AMPKalpha-PPAR-gamma in 3T3-L1 adipocytes and antiproliferatory effect through AMPKalpha-COX-2 in cancer cells. *Journal of Agricultural and Food Chemistry*.

[32] Brattström C, Wilczek H, Tydén G, Böttiger Y, Säwe J, Groth C-G (1998). Hyperlipidemia in renal transplant recipients treated with sirolimus (rapamycin). *Transplantation*.

[33] Brattström C, Wilczek HE, Tydén G, Böttiger Y, Säwe J, Groth CG (1998). Hypertriglyceridemia in renal transplant recipients treated with Sirolimus. *Transplantation Proceedings*.

[34] Roland M, Gatault P, Doute C (2008). Immunosuppressive medications, clinical and metabolic parameters in new-onset diabetes mellitus after kidney transplantation. *Transplant International*.

[35] Lamming DW, Ye L, Katajisto P (2012). Rapamycin-induced insulin resistance is mediated by mTORC2 loss and uncoupled from longevity. *Science*.

